# Independent Component Analysis (ICA) based-clustering of temporal RNA-seq data

**DOI:** 10.1371/journal.pone.0181195

**Published:** 2017-07-17

**Authors:** Moysés Nascimento, Fabyano Fonseca e Silva, Thelma Sáfadi, Ana Carolina Campana Nascimento, Talles Eduardo Maciel Ferreira, Laís Mayara Azevedo Barroso, Camila Ferreira Azevedo, Simone Eliza Faccione Guimarães, Nick Vergara Lopes Serão

**Affiliations:** 1 Department of Statistics, Federal University of Viçosa, Viçosa, Minas Gerais, Brazil; 2 Department of Animal Science, Federal University of Viçosa, Viçosa, Minas Gerais, Brazil; 3 Department of Exact Sciences, Federal University of Lavras, Lavras, Minas Gerais, Brazil; 4 Department of Animal Science, Iowa State University, Ames, Iowa, United States of America; Tianjin University, CHINA

## Abstract

Gene expression time series (GETS) analysis aims to characterize sets of genes according to their longitudinal patterns of expression. Due to the large number of genes evaluated in GETS analysis, an useful strategy to summarize biological functional processes and regulatory mechanisms is through clustering of genes that present similar expression pattern over time. Traditional cluster methods usually ignore the challenges in GETS, such as the lack of data normality and small number of temporal observations. Independent Component Analysis (ICA) is a statistical procedure that uses a transformation to convert raw time series data into sets of values of independent variables, which can be used for cluster analysis to identify sets of genes with similar temporal expression patterns. ICA allows clustering small series of distribution-free data while accounting for the dependence between subsequent time-points. Using temporal simulated and real (four libraries of two pig breeds at 21, 40, 70 and 90 days of gestation) RNA-seq data set we present a methodology (ICAclust) that jointly considers independent components analysis (ICA) and a hierarchical method for clustering GETS. We compare ICAclust results with those obtained for K-means clustering. ICAclust presented, on average, an absolute gain of 5.15% over the best K-means scenario. Considering the worst scenario for K-means, the gain was of 84.85%, when compared with the best ICAclust result. For the real data set, genes were grouped into six distinct clusters with 89, 51, 153, 67, 40, and 58 genes each, respectively. In general, it can be observed that the 6 clusters presented very distinct expression patterns. Overall, the proposed two-step clustering method (ICAclust) performed well compared to K-means, a traditional method used for cluster analysis of temporal gene expression data. In ICAclust, genes with similar expression pattern over time were clustered together.

## Introduction

Gene expression time series (GETS) analysis aims to characterize sets of genes according to their longitudinal patterns of expression, improving the understanding of the biological processes and regulatory mechanisms of genes that share similar expression profiles over time [1]. Specifically, in GETS studies, given the large number of genes evaluated, such as those using RNA-seq data, summarization of expression profiles into a small number of clusters that include genes with similar expression over time is a typical and useful strategy to deal with the high dimensionality of GETS data sets.

In general, the methods used for gene clustering can be split into two groups. One composed by traditional methods, such as hierarchical clustering [2] and k-means [3] methodologies, which consider observations at each time as independent variables for the clustering process. K-means optimizes the variance of the clusters, whereas hierarchical methods minimize the radius of the clusters. In general, k-means outperforms hierarchical clustering, since is likely to be a poor choice for further computational analysis of the resulting clusters. [4, 5]. Although these methods are of easy application and interpretation, they have as disadvantage the fact that the temporal dependence between time-points is not taken into account in the clustering process. In the other group we have the so called model-based cluster methods [6, 7], which require normality of the data, and cluster membership is decided based on maximizing the likelihood of data points given the cluster models [1]. However, RNA-seq data, which has discrete distribution (counts of reads), is not suitable to be used in these methods that assume normality of the data [8]. In general, GETS analysis present small number of the temporal expression measures.

The application of model-based methodologies in RNA-seq data (discrete variable) presents some challenges, such as the lack of normality (assumed in several models) and the small number of temporal observations (small series), which leads to poor estimation of the effects used in the clustering process. In this context, a methodology that can be used to cluster small series of distribution-free data while accounting for the dependence between subsequent time-points should be used for temporal analysis RNA-seq data. The Independent Component Analysis (ICA) [9] is a statistical procedure that uses a transformation to convert raw time series data into sets of values of independent variables, which can be used for cluster analysis to identify sets of genes with similar temporal expression patterns. ICA is a powerful methodology, especially when traditional methods, such as principal component and factor analyses, are ineffective, since these can still find intrinsic factors that support the observational data [9].

In summary, in this paper we propose a methodology named ICAclust that jointly considers ICA and a hierarchical method for clustering temporal RNA-Seq data. The proposed methodology was applied to GETS using temporal simulated and real RNA-seq data.

## Material and methods

### Independent Component Analysis

Independent Component Analysis (ICA) uses the existence of independent factors (latent variables) in multivariate data and decomposes an input data set into statistically independent components [9].

Assume **Y** = (**y**_1_, **y**_2_, …, **y**_m_)^T^ as the random vector. ICA approach assumes that **Y** can be modelled as linear combination of n independent components **S** = (**s**_1_, **s**_2_, …, **s**_n_)^T^, with some matrix of unknown coefficients **A** = [a_ij_], named mixing matrix, **Y**_m×N_ = **A**_m×n_ ∙ **S**_n×N_.

Considering the observed data set, y_ij_ corresponds to the mean expression value (considering multiple replicates) at time j for the i^th^ series (gene). Therefore, each serie y_i_ is decomposed into a linear combination given by y_i_ = a_i1_s_1_ + a_i2_s_2_ + ⋯ +a_ik_s_n_, for every i = 1, 2, …, m, so that each series is represented by the coefficients of each independent component of the mixture.

ICA has been used for dimension reduction [10]. This possibility is specially interesting for situations approaching high dimensional problems. Aiming reduction of dimensionality, a number k ≤ n of independent components (IC) can be selected by using principal component analysis (PCA) as pre-processing for ICA, so that, **Y**_**m×N**_ ∝ **A**_**m×k**_ ∙ **S**_**k×N**_, where **A** can be approximated by the product **KR**, where **K** is an orthogonalization matrix and **R** the matrix that maximizes the statistical independence of the columns of the matrix **S**. However, because of the low number of temporal observations in GETS analysis, we should use k = m.

To verify the significance of independence hypothesis between the independent components the non-parametric Hoeffding test [11] was performed. Hoeffding test computes D statistics, which represents the distance between F(x,y) and G(x) H(y), where F(x,y) is the joint cumulative distribution function (CDF) of X and Y, and G and H are marginal CDFs.

### Two-step algorithm (ICAclust) for clustering genes with similar gene expression patterns

**Fig 1:**
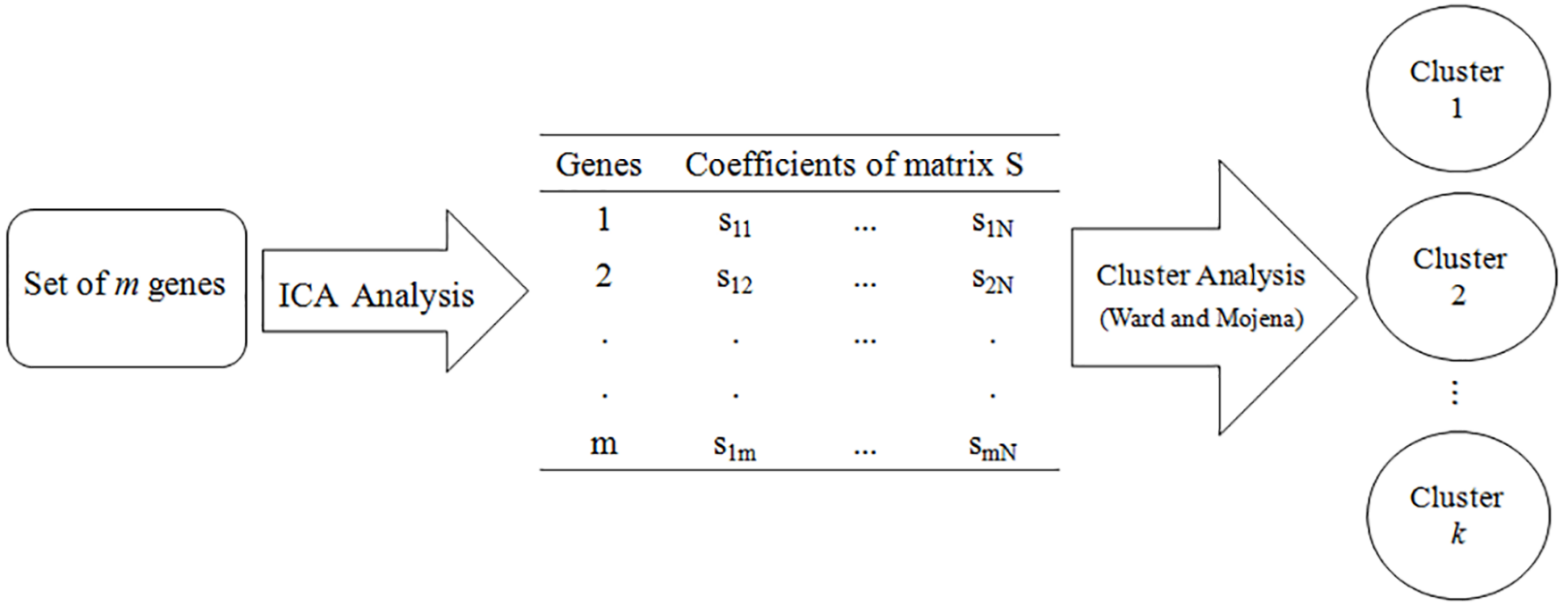
Flowchart summarizing the ICAclust approach.

Gene clustering was performed using a two-step approach, called ICAclust, in which ICA is initially applied to convert raw time series data, **Y**_m×N_ = (y_ij_) into statistically independent components. Thus, the new data set, composed by elements of matrix of independent component **S**, can be used as input variables in hierarchical cluster analysis using Ward’s method [12], with the number of cluster being defined by Mojena's criterion [13]. In the Ward's method, the goal at each stage of clustering is minimize the increment of the within-group error sum of squares by combining two individuals. Considering two groups, A and B, the increment is defined by ${\text{I}}_{AB}=\frac{{\text{n}}_{\text{A}}{\text{n}}_{\text{B}}}{{\text{n}}_{\text{A}}+{\text{n}}_{\text{B}}}{\left({\bar{\text{y}}}_{\text{A}}-{\bar{\text{y}}}_{\text{B}}\right)}^{\text{T}}\left({\bar{\text{y}}}_{\text{A}}-{\bar{\text{y}}}_{\text{B}}\right)$, where n_A_ represents the number of individuals of A, n_A_ represents the number of individuals of B, ${\bar{\text{y}}}_{\text{A}}$ and ${\bar{\text{y}}}_{\text{B}}$ are vectors giving the means of the variables of groups A and B, respectively. Mojena's criterion, suggests that one should select the number of groups corresponding to the first stage in the dendrogram satisfying the condition: $\alpha_{\text{j}+1}>\bar{\alpha }+{\text{cS}}_{\alpha }$, where α_0_, α_1_, …, α_n−1_ are the fusion levels corresponding to stages with n, n-1, …, 1 clusters. The terms $\bar{\alpha }$ and S_α_ are the mean and standard error of α′s, respectively; and c is a constant equal to 3.50 [13]. Fig 1 shows a scheme of the proposed method ICAclust. The resulting clusters from this analysis contain genes with similar expression patterns over time.

### Real data

GETS analyses were performed with four libraries of two pig breeds (Piau and commercial breed) at 21, 40, 70 and 90 days of gestation. Animals were raised at the Pig Breeding Farm from Federal University of Viçosa, Brazil. Pregnant gilts were euthanized at each day of gestation following the procedures described at [14]. For every breed, three sows were used for each time point and embryos/fetuses were collected (four library per breed). *Longissimus dorsi* muscle samples were collected from the embryos/fetuses, except for those at 21 days post gestation, in which the whole embryo was used. The collected material was placed in tubes with RNAlater solution (Ambion, Carlsbad, CA, USA) and stored at 4°C overnight and at -80°C prior to RNA isolation. The procedures for obtaining the embryos and fetuses were approved by the Ethics Committee for Animal Use at UFV (protocol no. CEUA-UFV 85/2013), in accordance with current Brazilian federal legislation.

Total RNA was isolated with RNeasy Mini Kit (Qiagen, Valencia, CA, USA). The total concentration of RNA was estimated in a spectrophotometer NanoVue TMPlus (GE Healthcare, Freiburg, Germany) and quality checked at the Agilent 2100 Bioanalyzer (Agilent Technologies, Palo Alto, CA, USA). rRNA were depleted using RiboMinus Eukaryote kit (Invitrogen, Carlsbad, CA). Then, RNA was fragmented by enzyme RNAse III, followed by purification and cDNA synthesis. Resulting samples were used for whole transcriptome library preparation for sequencing in SOLiD^™^ v.4 platform (Life Technologies Corporation, Carlsbad, CA, USA). RNA sequencing and all RNA processing procedures were performed using protocols and kits (SOLiD^™^ Total RNA-Seq) as recommended by Applied Biosystems. RNA sequencing was performed at the Research Center René Rachou (BH/MG), Minas Gerais, Brazil.

The data were visualized with fastQC and treated with Prinseq-Lite (v. 0.20.4; [15]. Reads were mapped by Bowtie software using *Sus scrofa* build 10.2 (Sscrofa10.2) as reference. After that, transcripts that had at least ten mapped reads across all the libraries were selected for subsequent analyses. On average, 36.75 million reads were obtained per library.

The proposed method ICAclust was applied to 458 genes that presented differential expression between breeds (FDR < 0.05) by empirical Bayesian approach based on posterior probabilities using the R package baySeq [16].

### Application to simulated data

We evaluated the proposed clustering method performance using simulated datasets, each one with 458 genes divided into 6 clusters. RNA-seq quantification is based on read counts (discrete variable), and thus, count distributions such as Poisson or negative binomial are the usual choices to account for the biological phenomenon under study [17]. Additionally, since our problem approaches temporally dependent measures, a multivariate count data distribution seems appropriate for this situation. Therefore, gene expression levels were generated over 4 time points using a multivariate Poisson model with a heterogeneous first-order autoregressive covariance structure. The gene expression time series for each gene in each cluster was sampled from Y_ik_ ~ P(**λ**_k_, **Σ**_k_), where Y_ik_ is the time series (a vector with dimension 1 x 4) of gene i (i = 1,2,…,g_i_) in cluster k (k = 1,2,…,6), **λ**_**k**_ = [λ_1k_ … λ_4k_]^T^ is the rate of occurrence vector, which were sampled from $\lambda_{\text{tk}}\sim \text{N}\left(\lambda_{\text{t}},\sigma_{\lambda \text{t}}^{2}\right)$ and the correlation matrix **Σ**_k_ is given by:

$$\sum =\left[\begin{array}{cccc} \sigma_{\text{t}1} & \phi_{\text{k}} & \phi_{\text{k}}^{2} & \phi_{\text{k}}^{3}\\ & \sigma_{\text{t}2} & \phi_{\text{k}} & \phi_{\text{k}}^{2}\\ & & \sigma_{\text{t}31} & \phi_{\text{k}}\\ & & & \sigma_{\text{t}14} \end{array}\right],$$

where ϕ_1k_ is the autoregressive parameter and $\sigma_{\text{tk}}^{2}$ is the variance in each time.

The number of genes (458) and longitudinal points (i.e. 4 time points) were chosen based of the real dataset presented in the previous section. The number of clusters (i.e. 6), and number of genes in each cluster, as well as the values of **λ**_k_, and σ_tk_ were determined according to the results using the real data and will be presented later. For ϕ_1k_, values used in the simulation were obtained by averaging the estimates obtained from each resulting cluster k, i.e., $\phi_{1\text{k}}^{}=\sum_{\text{i}=1}^{{\text{I}}_{\text{k}}}{\hat{\phi }}_{1\text{k}}^{}/{\text{I}}_{\text{k}}$, where ${\hat{\phi }}_{1\text{k}}^{}$ is the estimate of ϕ_1k_ for each gene belonging to cluster k, and I_k_ is the number of genes in cluster k.

In order to compare the clustering method (ICAclust) presented here with a traditional clustering method, the simulated datasets were also analyzed using the, k-means algorithm [18]. The choice of k-means is due to its general use in temporal gene expression clustering [7, 8, 19] and for its overall better performance over hierarchical clustering [4, 5].

The comparison between ICA clustering methodology and k-means was evaluated by mean correct classification rate (CCR), which was computed as the ratio between the numbers of genes clustered into the true cluster (from simulation) and the total number of genes over 10 replicated datasets. It is important to emphasize that, unlikely the proposed method, the number of clusters need to be defined prior to analysis in k-means. Since, in general, the number of clusters is unknown, we simulated the data using different number of clusters (k = 2, 3, 4, 5, 6 and 7). For ICAclust, we used different values of the Mojena’s constant (c = 2.25, 2.50, 2.75, 3.00, 3.25, 3.50 and 3.75) to evaluate their effect on the clustering process. These values were chosen aiming to expand the optimal clustering range (2.77–3.50) suggested by [13], and thus, being more conservative in our comparisons.

### Computational features

The simulation process was carried out with the function *gen*.*PoisBinOrd* of the PoisBinOrd R package (http://R-cran.org). The proposed method, denoted by ICAclust, was implemented in R, through the combination of fastICA [20], hclust R functions and the Mojena’s criterion. The R scripts for implementation of the proposed clustering method, and the real and simulated data sets are freely accessible at https://zenodo.org/record/571134#.WQsuLca1vIU.

## Results

### Real data

**Fig 2:**
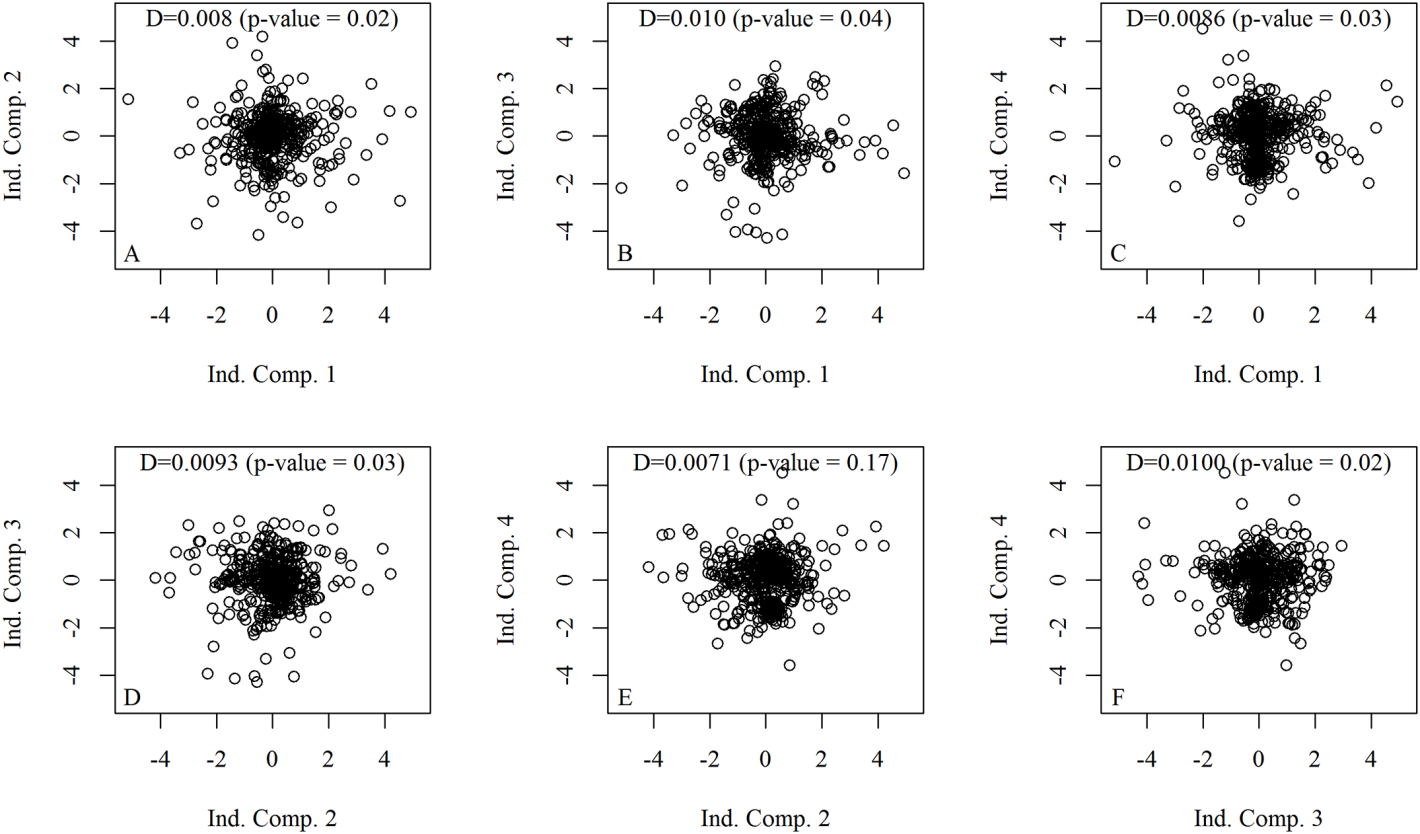
Scatterplots based on independent components, D statistics of Hoeffding and their associated p-values. The six scatterplots used to visualize relationships between the four independent components are represented in figures A to F.

The six scatterplots used to visualize relationships between the four independent components, D statistics of Hoeffding and their associate p-values are shown in Fig 2. As expected, the data showed a random pattern indicating absence of any association. Overall, p-values were greater than 0.01.

**Fig 3:**
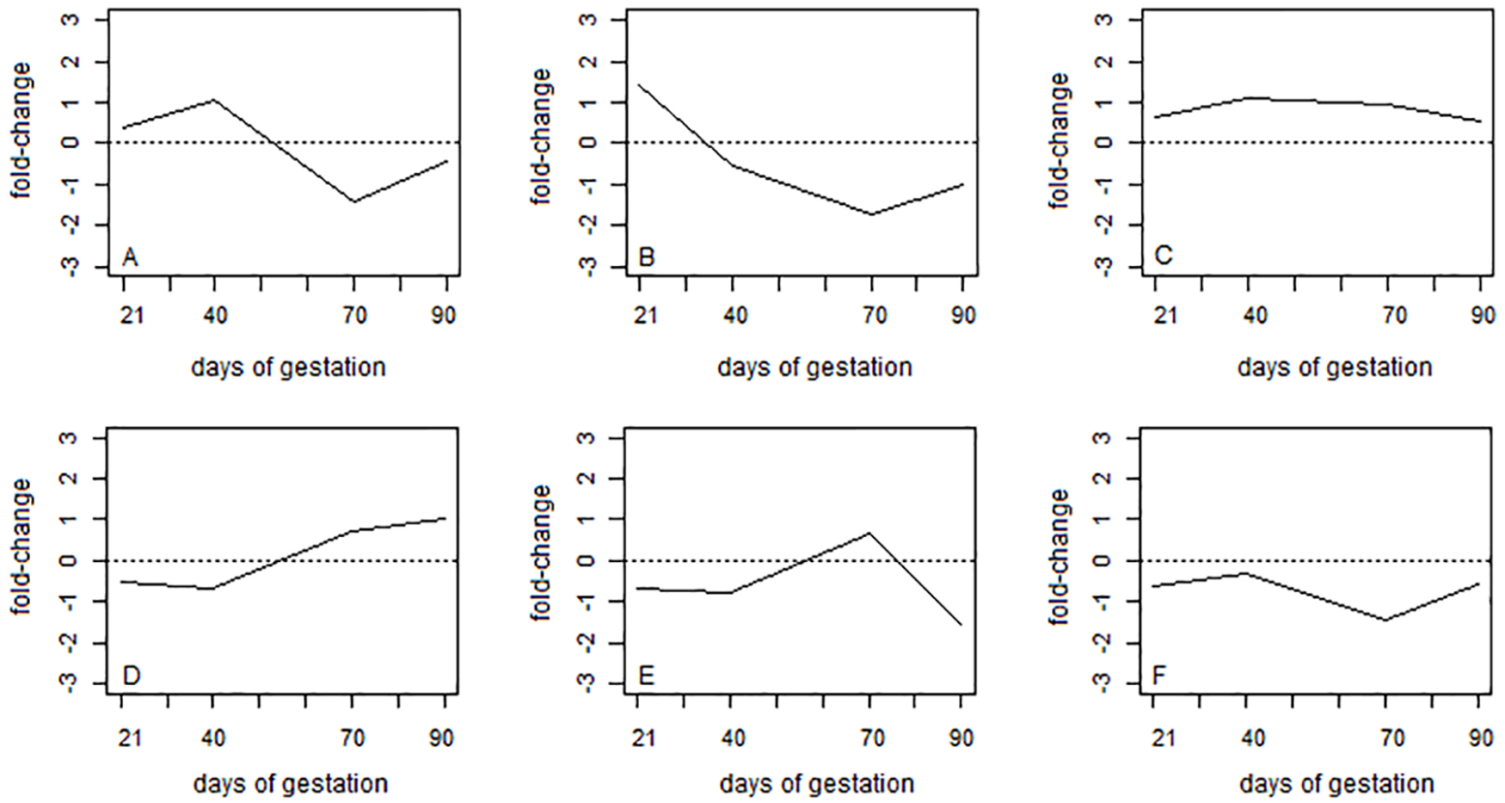
Time series average expression of six gene clusters found by the ICAclust. Fold-change = log_2_(Piau/Commercial). The six clusters are represented in figures A to F.

Using the four independents components as variables, genes were grouped into six distinct clusters (clusters A to F) with 89, 51, 153, 67, 40, and 58 genes each, respectively. In general, it can be observed that the 6 clusters presented very distinct expression patterns (Fig 3). Fold-change is presented as the log_2_ of the ration between the reads in the Piau and Commercial breeds. Among the various differences, it can be observed that the genes that make up groups A and D had opposite average expression pattern across time. While Piau had greater overall expression than Commercial animals at 21 and 40 days of gestation in cluster A (Fig 3A), the opposite trend was found at days 70 and 90 of gestation in cluster D, where Commercial animals had greater expression than Piau. Genes belonging to the second cluster (Fig 3B) presented greater expression values in the Piau breed only at the beginning of gestation (i.e. 21 days). However, genes belonging to cluster C (Fig 3C) had greater expression in the Piau breed at all time points. In contrast, genes in cluster F had greater expression in the Commercial breed throughout the whole gestation period (Fig 3F). Furthermore, genes belonging to cluster E presented higher expression in Piau compared to Commercial only at 70 days of gestation (Fig 3E).

### Simulated data

**Fig 4:**
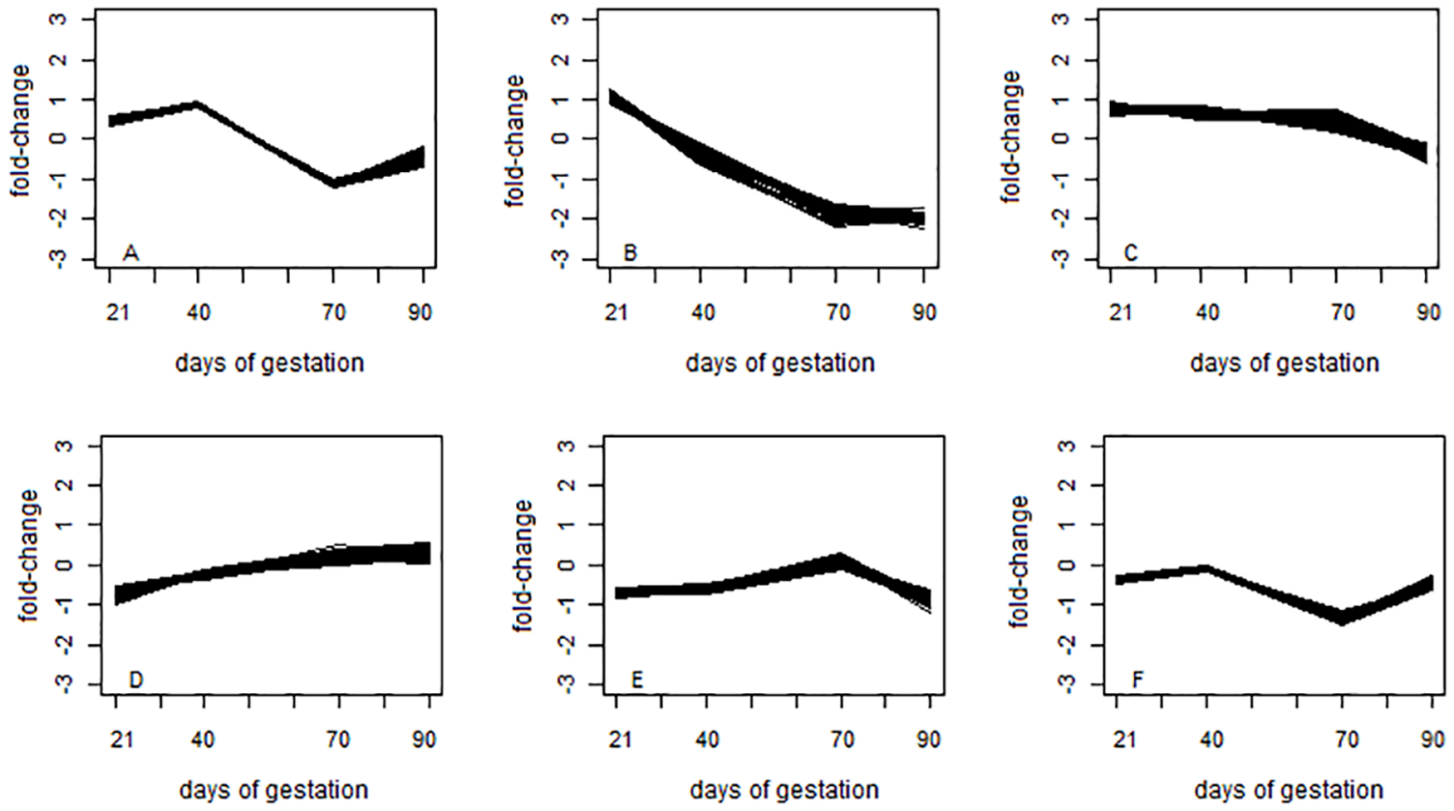
Average expression profile of over ten simulated data set considering the number of clusters determined according to the results using the real data. Fold-change = log_2_(Piau/Commercial). The six clusters are represented in figures A to F.

Ten replicates of gene expression profiles were simulated to compare the ICAclust and k-means methodologies. The average of expression profiles across all replicates is presented in Fig 4. The simulated data set presented similar temporal expression pattern to those clusters obtained in the real data analysis, showing that the simulation process was able to capture the same temporal relationship presented in the real data.

**Fig 5:**
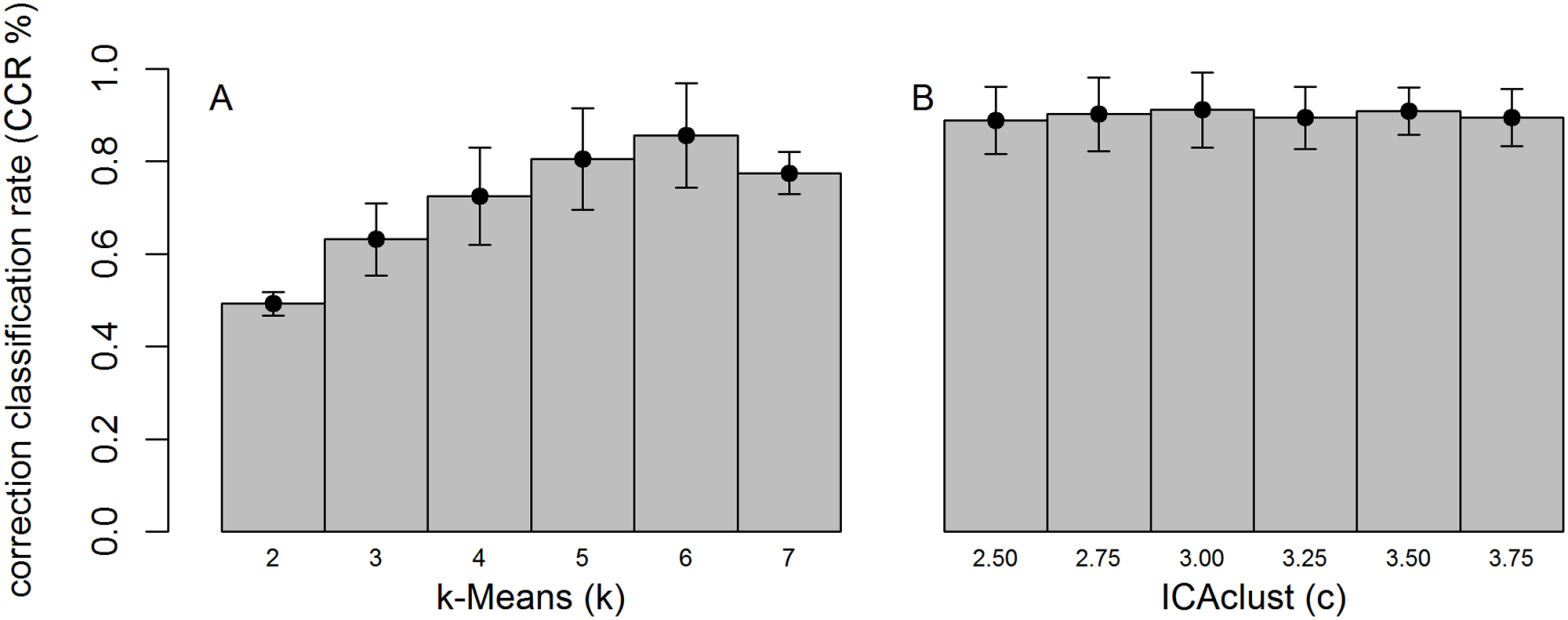
Average correct classification rate (CCR, %) for each clustering method across 10 replicates. The performance of k-means and ICAclust clustering methods are represented, respectively in figures A and B. Error bars represent the CCR standard deviation of 10 replicates.

Performance of the clustering methods based on the simulated data is presented in Fig 5. Correct classification rate (CCR), which considers the ratio between the numbers of genes clustered into the true cluster (from simulation) and the total number of genes, was used for evaluation over the 10 replicated datasets. As depicted in Fig 5A, k-means clustering had a lower CCR compared to ICAclust in all cases. The k-means method presented the highest average CCR (86%) when the number of clusters specified for analysis was the same as the number of simulated clusters (i.e. 6), thus, representing the best case scenario for this method. Furthermore, CCR values decreased as the number of clusters specified for the analysis moved away from 6.

On average, all ICAclust results had great CCR than k-means, and ranged from 89% to 92%, for c between 2.50 and 3.75, respectively (Fig 4B). Moreover, ICAclust presented, on average, an absolute gain of 5.15% over the best k-means scenario (k = 6). Considering the worst scenario for k -mean (k = 2), the gain was of 84.85%, when compared with the best ICAclust result (c = 3.00). Differently than for k-means, which requires to have the number of clusters (k) defined to perform cluster analysis, ICAclust uses Mojena’s criterion to determine the number of clusters automatically at end of the clustering process, and thus, increases the CCR.

**Fig 6:**
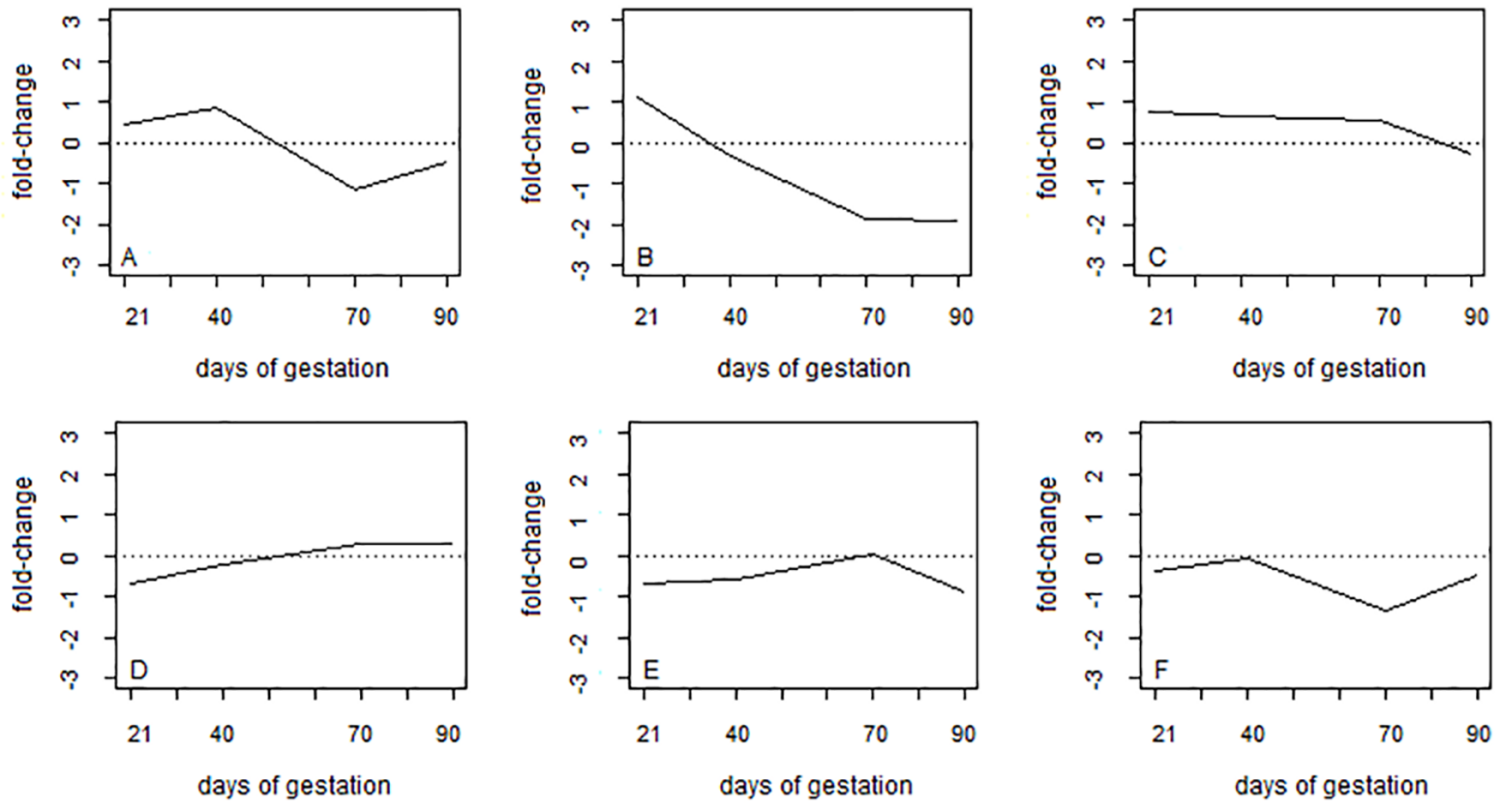
Time series average expression of six gene clusters found by the ICAclust considering values for c between 2.50 and 3.75. Fold-change = log_2_(Piau/Commercial). The six clusters are represented in figures A to F.

The modal number of clusters identified by ICAclust was 6 over all replicates and Mojena’s constant (c) values. Time series average expression of clusters, considering the modal number of clusters (k = 6), found by ICAclust is presented in Fig 6. The patterns presented in this figure are similar to those obtained from the simulated data (Fig 6), indicating that this methodology was able to create the same results as those using the real data (Fig 4).

**Fig 7:**
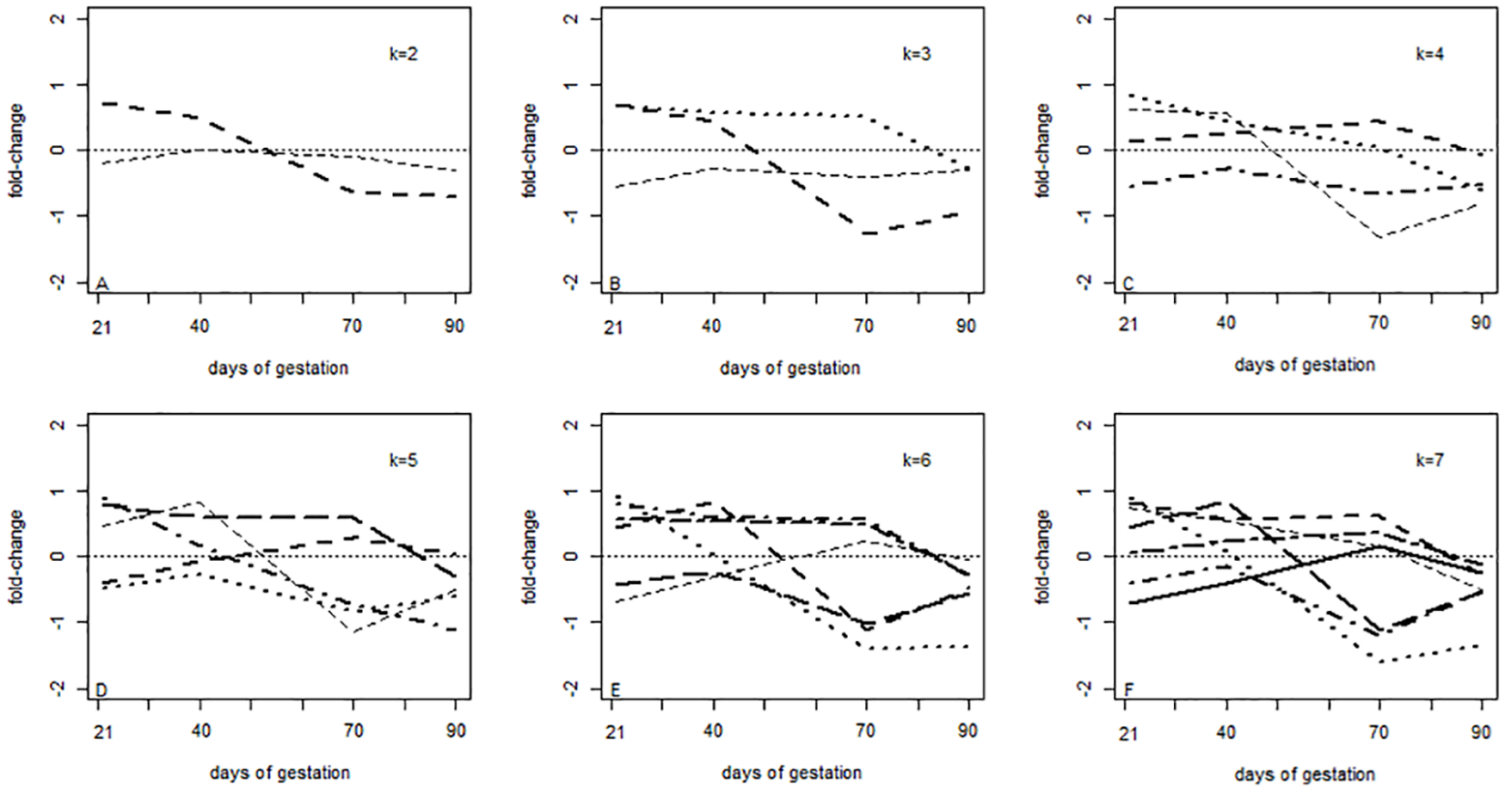
K-means clusters. Fold-change = log_2_(Piau/Commercial). Time series average expression of clusters found by k-means considering k = 2, 3, 4, 5, 6 and 7 are represented, respectively, in figures A to F.

Time series average expression of clusters found by k-means considering k = 2, 3, 4, 5, 6 and 7 are presented in Fig 7.

The correct simulated pattern was only observed when the correct number of groups (k = 6) was informed for k-means method. However, in real life, the correct number of clusters is unknown. When a lower number of clusters is considered (k < 6) in k-means, unique gene expression patterns can hidden. On the other hand, when the number of clusters used for analysis is higher than true value, some gene expression patterns can be split into two new groups.

Although the simulated data set was generated according to the results from the real data analysis, the comparison between the proposed ICAclust and the traditional k-means methods is reasonable. While ICAclust performed well using a traditional hierarchical method without losing information about the relationship between observations, the traditional k-means method did not account for this dependence, leading to worse results compared to those obtained by ICAclust.

## Discussion

In this paper we have presented the ICAclust methodology, which can be used to decompose RNA-seq data into statistically independent components and to group genes into mutually exclusives clusters.

Independent Component Analysis (ICA) decomposes an input data set into statistically independent components. In the last decade, ICA approach was proposed as PCA, to reduce the dimensionality of the data [10]. However, differently than for PCA, where components are independent only in the presence of multivariate normality of the input variables, in ICA we can obtain statistically independent components even in the absence of multivariate normality. Therefore, ICA seems ideal for data with mixed distributions, such as those found in high-dimensional and non-normal RNA-seq data sets. Since ICA naturally takes the temporal dependency into account through its underlying model when decomposing variables [21], ICA gives the opportunity to quickly generate independent components and then group them based (e.g. genes) based on the temporal dependence among them. In our methodology, the number of latent variables (i.e. independent components) is equal to the number of samples in gene expression data, i.e., 100% of original information is used in the clustering process. The small number of RNA-seq samples does not present any problems, as ICA has been successfully used in many studies with microarray data and cluster analyses [22]. In addition, differently than the most clustering methodologies, our method outputs the number of the clusters automatically at the end of clustering process. ICAclust methodology is simpler and quicker than based-model methods [6, 7], specifically those using a Bayesian approach, since these require evaluating convergence of the chains.

Although several advantages of ICAclust have been reported here, one possible disadvantage is that this method is not model-based. Some model-based approaches have been specially indicated for time course RNA-seq studies [23], such as the autoregressive time-lagged regression and hidden Markov models. However, when working with short gene expression time series, as in the present study (only four temporal measures), the model-based methods can lead to poor clustering performance [24].

One interesting point to be exploited under a gene clustering approach is to examine how the genes are assigned to clusters as the number of clusters increases. Schonlau [25] proposed a relevant method denominated “clustergram”, which enables to visualize the clustering formation and give insight on the optimal number of clusters. Since we used the Mojena criterion to identify this number, one future implication might be to update the ICAclust to provide information requested for “clustergram” implementation. Finally, with the rapid increase in the size of high-throughput genomic data, other efficient algorithms, such as MapReduce [26] and trie trees [27], could be considered in the future to improve the computational performance for read alignment.

## Conclusions

The proposed two-step clustering method (ICAclust) performed well compared to k-means, a traditional method used for cluster analysis of temporal gene expression data. In ICAclust, genes with similar expression pattern over time were clustered together. Compared to k-means method, ICAclust methodology present some advantages: (i) the dependence between observations are take account in the clustering process through of independent components that are linear combinations of original variables; (ii) it is not necessary to define the number of the clusters prior to analysis, as these are obtained automatically using Mojena’s criterion; (iii) ICAclust does not make any assumptions about the data distribution, i.e., it can be used for discrete data such as RNA-seq data; and (iv) it performed well to small number of temporal observations. However, more studies using different RNA-seq data sets are needed to further validate results found in this study.
